# Towards a More Reliable Identification of Non-Conformities in Railway Cars: Experiments with Mask R-CNN, U-NET, and Ensembles on Unbalanced and Balanced Datasets

**DOI:** 10.3390/s24237642

**Published:** 2024-11-29

**Authors:** Eduardo Carvalho, Bruno Ferreira, Ana Claudia Gomes, Camilo Gonçalves, Giovanni Dias, Renato Torres, Gustavo Pessin

**Affiliations:** 1 Instituto Tecnógico Vale Desenvolvimento Sustentável, Belém 66055-090, Brazil; 2 SENAI Innovation Institute for Mineral Technologies, Belém 66035-080, Brazil; bruno.isi@senaipa.org.br (B.F.); claudia.isi@senaipa.org.br (A.C.G.); 3 Institute of Technology (ITEC), Postgraduate Program in Electrical Engineering (PPGEE), Federal University of Pará, Belém 66075-110, Brazil; camilolgon@gmail.com; 4 Vale S.A., Maranhão 65085-580, Brazil; giovanni.dias@vale.com; 5 Institute of Exact and Natural Sciences (ICEN), Postgraduate Program in Computer Science (PPGCC), Federal University of Pará, Belém 66075-110, Brazilgustavo.pessin@itv.org (G.P.); 6 Instituto Tecnógico Vale Mineração, Ouro Preto 35400-000, Brazil

**Keywords:** computer vision, convolutional neural networks, image classification, image segmentation

## Abstract

In every business, equipment requires repair services. Over time, equipment wears out; however, with well-conducted and guided maintenance, this degradation can be controlled, and failed equipment can be restored to operational status. Preventive maintenance allows this concept to be applied, given the great advantages for large companies in reusing equipment and machinery, always putting the worker’s health and safety first. Rail transport has several pieces of equipment that can be reused if they are in a regular and well-defined maintenance cycle. In this sense, this article sought to create a method using real data for identifying cracks in wagons. Through the use of computer vision algorithms to prepare the data, along with several machine learning classification algorithms to locate cracks in train cars, the classification used properly annotated images and obtained great results, with a best case 98.10% hit-rate when wagons had a crack problem.

## 1. Introduction

Cargo transportation by train in mining operations is the primary logistical activity in this category. During this activity, several defects can occur, ranging from loose parts to problems arising in the carts [1]. Even if a maintenance system is in place to assess breakdowns or equipment failures, a train derailment can lead to astronomical costs for mining companies [2], resulting in millions of dollars in expenses.

Concerning maintenance management, individuals typically bear the responsibility for assessing the feasibility of using specific parts on wagons for new trips or determining whether a wagon needs to be taken out of service for maintenance. However, relying on human involvement in this process poses risks, both for the maintenance itself and for the individuals conducting the assessments. To mitigate these risks, an increasing number of companies are adopting computer vision solutions to inspect certain situations and parts [3]. Despite its widespread usage, automatic inspection through image analysis is not without challenges. The identification procedure often involves several stages, including image acquisition, preprocessing, feature extraction, and, finally, classification.

The feature extraction phase in this type of system faces the challenge of specialist approval, since prior knowledge of what constitutes a defect is necessary. In this paper we show examples of a crack in a wagon, and examples of a part of a wagon with no issue. To eliminate the reliance on human judgment in the process, a classification system for structural problems on railway cars can incorporate machine learning algorithms. This approach removes the decision-making burden from the maintainer and, with their assistance, focuses solely on creating a database to evaluate the objective of the task. The use of convolutional neural networks (CNN) and their variants has proven to be a widely adopted technique for image classification [4,5].

One challenge in machine learning, including with CNNs, is the ability to learn models from unbalanced datasets, which often occurs during the acquisition of images in real-world scenarios. For instance, the shear pad (hereafter referred to as PAD) is one of the crucial components of a wagon that requires inspection [6]. However, large organizations possess a substantial fleet of wagons, comprising various wagon models. Furthermore, not all wagons are equipped with a PAD, and only a very small percentage of them exhibit defects. In essence, an automatic wagon inspection system must handle a multi-class dataset with a significantly high imbalance rate.

Another challenge in working with machine learning is the creation of a reliable database for conducting nonconformity checks [7]. These databases are generated from real information, such as images. For instance, the superstructure is of significant importance when inspecting wagons. However, large companies often have a vast number of wagons, and they operate at least two different types of wagons on a daily basis for transporting ores. This paper presents results solely for one type of wagon.

Works utilizing variations of U-Net networks have found applications in various fields. While this method initially began with medical applications years ago [8], its versatility has expanded to other areas. One such area is transportation, where these adaptations can be applied in real-world scenarios, as well as simulations [9,10,11].

In this context, this work aimed to utilize machine learning techniques, such as multilayer perceptron (MLP), CNN, Mask R-CNN, U-Net, and an ensemble of these different methods, to accurately classify the condition of wagon components through images. Specifically, we employed a preprocessed database to aid our classifications and evaluated the performance of these algorithms using two different databases.

The objectives of this work aimed to contribute to two main points: (i) the development of a workflow proposal for identifying non-conformities in train cars, and (ii) evaluation of the method proposed in the previous objective, with different machine learning algorithms on two different types of databases: one unbalanced and one balanced. We anticipated that by addressing these two questions, we could develop a new method for preparing images of train cars to identify defective parts, as well as proposing a way to classify cracks using different methods.

The remainder of this paper is organized as follows: the next section presents related works associated with our method, followed by a brief description of the real-world problem with which our method was tested in the subsequent section. Section 4 describes the methodology used for data collection, as well as the methodology for classification algorithms, which we used to evaluate our databases. Section 5 presents our experimental results, while Section 6 provides a comparison of the results obtained using other methodologies and classification algorithms. Finally, the last section concludes the paper by presenting our plans for future work.

## 2. Related Work

This section describes relevant works from the literature, such as the work of Gonçalves et al. [12], which explored the use of HOG descriptors for identifying train parts and creating a database consisting of three classes of PADs. In their study, images were extracted using descriptors combined with SVM. The objective was to determine the optimal distance within a large-scale image for locating the PADs. To achieve this, the classifier identified the best bounding boxes in three intervals, allowing for the removal of the PADs from the image.

The work presented by [6] focused on utilizing a discrete Fourier transform (DFT) in the spatial representation of railway car components, to develop an automatic detector for defective parts using convolutional neural network (CNN) classification. Their findings demonstrated the classification of PADs using metrics such as accuracy, precision, recall, and F1-score, as well as an accuracy boxplot. The results indicated that the use of DFT improved the CNN classification accuracy by 1.04%. This work serves as a practical application based on the database created in the study by [12].

**Figure 1:**
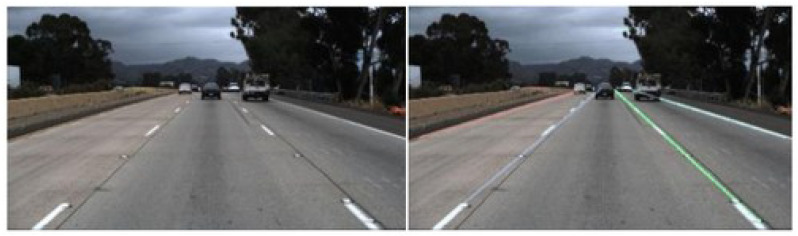
Lane detection for a highway [11].

An example of work in the transportation field utilizing U-Net was proposed by [11]. In their study, the authors aimed to identify the boundaries of car circulation lanes through image analysis. They employed the U-Net algorithm for lane segmentation. The authors introduced group-by-group convolution and depth-wise separable convolution into the backbone network, which simplified the branches of the network. They also incorporated convolution into the enhanced path network with a multi-level skip connection structure, to preserve coarse-grained semantic feature information. The full-scale skip connection fusion mechanism of the decoder was retained, enabling the capture of both fine-grained and coarse-grained semantic features at full scale. By introducing skip connections between the decoder and the encoder, the network enhances lane extraction, without increasing the receptive field size. The network’s ability to extract line features and contextual information improves the accuracy of lane line detection. Experimental results demonstrated that the improved neural network achieved excellent performance in detecting complex lane lines, effectively enhancing both the accuracy and time sensitivity of lane line detection. Figure 1 showcases the image segmentation results achieved using U-Net.

The work of [13] showed the use of hyperspectral images for target selection, using only one spectrum. In the paper, they detailed the creation of a background dictionary that consisted of identifying only one atom target. The proposed method was compared with other techniques shown in the article and had the best performance. The work of [14] used labels to identify and recreate regions in multi-spectral images and classify them according to their characteristics, according to the model proposed in the article, which had relevant results for a part of the terrain images using three different databases from different sensors.

The work of [15] conducted a study on the 2D modeling of CNNs to identify micro-cracks in train gears. In the study, the authors indicated that the trained model detected these cracks with 92% accuracy. In this context, it is essential to mention that, along with the work of [15], there are correlations with other wagon components, such as checks for various gear types (utilizing images), other components (wheels and brakes), and even other forms of analysis.

The paper by [16] focused on inspecting fatigue in a wheel axle. The study identified fractures caused by plane deformation, the direct consequence of inadequate maintenance of the car parts. Two types of tests were conducted to determine the occurrence of cracks and assess the operational condition of the wagons. However, the results consistently concluded that replacing the parts was necessary to prevent accidents. The primary tests performed in this study were tension tests, involving the application of longitudinal and transverse forces to the wheel axles. Additionally, chemical testing using optical emission spectroscopy (OES) was conducted on these axles [17], similarly to that of [16], focusing on studying fatigue in wagon axles. In addition to mechanical calculations, their work included thermal testing of the structures. In a simulated environment, both situations were tested, revealing that the thermal indications of the braking points of the wheels had the highest incidence of problems. Furthermore, the vertical angular force on the wheels emerged as the most severe mechanical issue observed in their tests.

The work by [18] addressed the identification of problems in their organization, to improve train brake maintenance management. The study took into account the optimal timing for brake replacement, considering the cost reduction for the company. It also determined the storage of brakes and their availability for scheduled exchanges. The authors concluded that significant savings of 25% could be achieved if the brake depot was readily available for immediate exchanges in train cars.

Peng and colleagues presented a method that focuses on addressing cracks resulting from brake wear on trains. The authors proposed a three-step analysis approach to examine these cracks. Firstly, a finite element model of the wagon wheel is utilized for all braking applications. Secondly, the stress intensity factor of the thermal cracks is calculated. Lastly, the authors employed the generalized Frost–Dugdale approach to model crack growth. Through simulations, the authors observed that vibratory forces were the primary cause of cracking problems during stages 2 and 3 of the testing process [19].

## 3. Statement of the Problem

In many railway companies, wagon maintenance is carried out by employees, who are tasked with evaluating several components within a given time frame. These components include the compression bar, triangle, bearing box adapter, and coupling support plate. Since wagons are crucial assets in railway operations, it is essential to ensure accurate and optimized maintenance practices. However, visual inspections conducted by humans pose risks to employees, as they often need to work in hazardous environments, such as being in close proximity to moving trains, to inspect these items [20].

To be more specific, our study focuses on a specific wagon component known as the superstructure. This metal component is susceptible to cracking due to the constant insertion and removal of ore. In this work, we specifically consider the left side of the superstructure, with a particular focus on the joints, which we refer to as pillars.

This study is part of a larger project aimed at developing an automatic inspection system for wagon components for the one of the largest mining company in the world, who operate approximately 2000 km of railroad tracks in Brazil. Rail transportation plays a crucial role in any mining operation, and the company operates one of the largest trains in the world, consisting of four locomotives and 330 wagons. The company transports iron ore through its railway network 24 h a day, 7 days a week.

Given the large quantity of wagons, various models, and the challenging operating environment, the superstructure component is prone to various types of damage. To address this, a camera system is employed to capture images, which are then processed using image processing and computer vision algorithms. The focus of this paper was to evaluate our method’s performance in classifying superstructures cracks based on the collected images. We utilized two different databases: one with a single class —crack, and another with two classes —crack and pillar. The methodology and experiments sections provide detailed information on how the tests were conducted.

## 4. Methodology for Identifying Cracks in Freight Trains

In this section, the methodology developed for the work will be presented, encompassing the data collection, data pre-processing, image transformations, creation of image masks, and definition of classifier algorithms.

The entire process was carried out on a Lenovo notebook, model IdeaPad Gaming 3i, with a Core I5-10300H processor, GeForce GTX 1650 video card with 4 GB, 8 GB of memory, and 256 GB of storage.

### 4.1. Collection and Preprocessing Methodology

**Figure 2:**
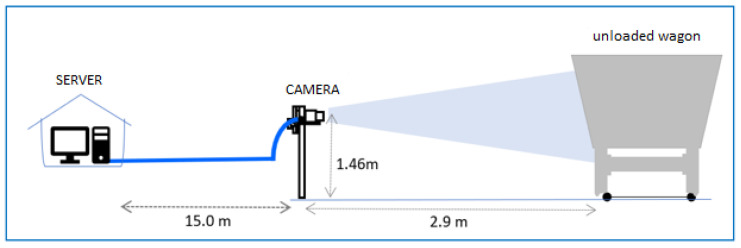
Distance of the camera to the railway car.

The methodology for collecting or acquiring images to identify cracks in train carts involves several phases. Firstly, a camera trigger device is activated based on the passage of the carts. Once triggered, the camera starts recording, and the footage is stored on a local server. As depicted in Figure 2, these recordings are named according to the date and time of the train’s passage. The camera installed at the collection site captures images with a fish-eye effect, which are later rectified using the camera’s calibration data. The image preparation process involves transforming the recorded footage, captured in 4k resolution, into Full HD resolution (1920 × 1080 pixels) to facilitate the training of the classification algorithm.

**Figure 3:**
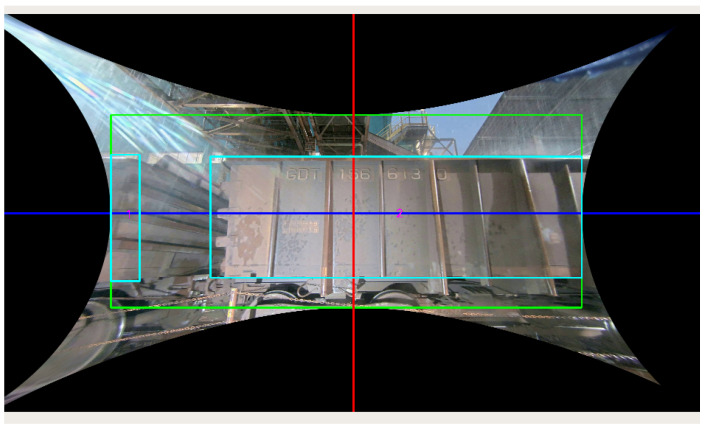
Removal of the eye-fish effect of the axis camera and the single-shot detector (SSD) applied to the railway car. In this effect removal, the green box delimits the new area of the new figure, the red lines are the center of the image on both axes, and finally the blue box are the superstructure delimitation’s.

The camera’s recording phase is followed by a detailed preprocessing stage. The first step in preprocessing was adapted from the work of Gonçalves et al. [12]. In this phase, histogram of oriented gradients (HOG) descriptors are applied to identify a structure (train drain) of the wagon’s truck. Using this information, a single-shot detector (SSD) segmentation algorithm is employed to calculate the edges of each wagon and assign them unique numbers. These numbers serve to identify and enumerate each group of wagons in a series of images captured from the video. By analyzing the classifier’s indications, it can be determined if the first wagon in a composition has a crack. Figure 3 illustrates an example of the SSD applied to a wagon, where the parameters for removing the fish-eye effect are given, and the measurements between wagons are indicated to aid in the identification and counting of wagons with or without issues.

The removal of the fisheye effect at this stage of the process relies on the bounding boxes present in Figure 3. The green bounding box is the new figure that will continue in the process flow, while the blue bounding box indicates the area of the wagon, as well as the count of the composition of wagons passing in front of the camera.

After the wagon detection phase, the images are extracted from the recorded video frame by frame and saved. To avoid having multiple identical images in the database, the images are cropped, and a sampling algorithm is applied based on the size of the images and the presence of the majority of the wagon, as identified during the detection phase.

With this final image in hand, the preprocessing steps of image collection and storage are completed. In the next stage, these images are prepared for the classifier. The intention of reducing the sizes of the wagon parts is to ensure that the classification step does not become computationally burdensome and to avoid potential issues of overfitting. This allows the individuals who inspect this particular part of the wagon to focus on other areas not covered by this work.

**Figure 4:**
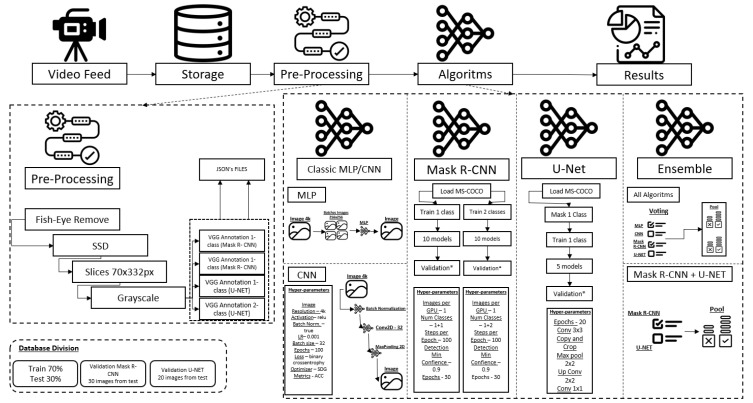
Information workflow throughout the entire process, from acquisition to final result. The asterisk marked in validation is as described in “Database Division”.

The overall methodology is presented in Figure 4. The Figure shows the complete flow of the methodology, from video capture to results. However, the pre-processing and classification algorithms are also detailed. The pre-processing was covered in this section, and the explanation of the algorithms will be detailed below.

### 4.2. Classification Methodology

The classification methodology involves several additional steps after the pre-processing stage, before the algorithms can provide information to a company employee about whether the wagon has a crack or not.

**Figure 5:**
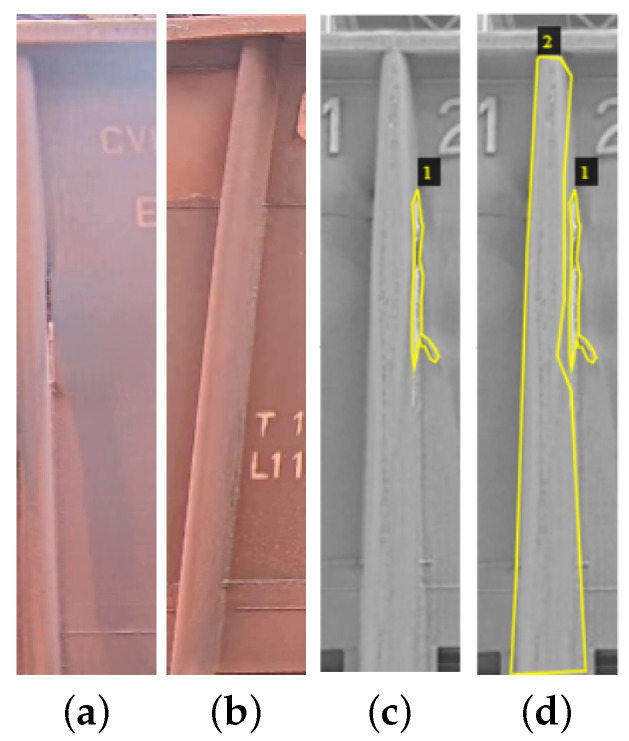
Examples of train images used throughout the defect image classification process. (**a**) Image with tear problem at the train joint; (**b**) profile without problems considered by the maintainers; (**c**) class one example; (**d**) class two example.

In this work, two databases were considered. The first database includes images where only cracks are identified, as shown in Figure 5c. These images were captured from video of wagons passing through the inspection area, and the areas containing cracks were annotated. The second database includes images where areas with cracks were identified based on reports from workers, representing approximately 95% of the areas with reported problems. These areas are depicted in Figure 5d.

#### 4.2.1. MLP Methodology

Before performing the pre-processing, a simple test was performed with the 4k images, running a simple MLP to verify whether the simplest neural network would be able to classify this problem.

To simplify the entrance for the MLP, a resizing of the images in 4k to the resolution 256 × 256 was implemented. Several problems were identified, which will be discussed later, but only one neuron was used for this test, as shown in Figure 4.

#### 4.2.2. CNN Methodology

**Figure 6:**
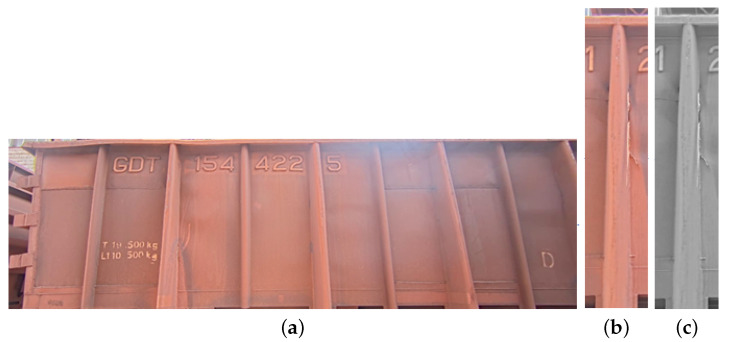
Pre-processing processes of images taken from the video acquisition of the left side of train cars. (**a**) Result of the SSD algorithm identifying the left side of a wagon; (**b**) 70 × 332 is the image size input to the classifier; (**c**) gray scale transformation for the classifier.

Due to the problems encountered in using only a simple MLP and due the nature of the images, as well as the success in classifying other defective parts with the CNN algorithm, the next step was to implement this technique, in conjunction with Figure 6, in image preprocessing to improve the base and create image classes as in Figure 5, in conjunction with Figure 6, in image preprocessing to improve the base and create image classes as in Figure 5, Following this step, the images have the shape as shown in Figure 6a, with a size of 1120 × 332 pixels. Subsequently, the images are further cropped to a smaller size, before being passed to the classifier, resulting in the image shown in Figure 6b, which has dimensions of 70 × 332 pixels. In Figure 6c, the image is transformed to grayscale to simplify the input for the neural network.

As seen in Figure 4, a CNN was implemented with 4k images, with a batch normalization layer, a convolution layer, and a max pooling layer, to result in images with crack problems.

In Figure 4, you can see the hyperparameters used for this classification, remembering that no pre-processing step was performed for this classifier.

#### 4.2.3. Mask R-CNN Methodology

The problems encountered by these two algorithms (MLP and CNN) led to the pre-processing carried out and illustrated in the last Section. In this sense, this work focused on the creation of two databases for the evaluation of the Mask R-CNN and U-NET algorithms.

The R-CNN Mask requires annotated images for classification purposes. In this case, the annotations were performed using the VGG Image Annotator (VIA) program, specifically version 2. This version allows images to be annotated with their areas of interest, and the corresponding labels are indicated in a JSON file.

These annotated images served as the training data for the Mask R-CNN, allowing it to learn to classify and detect cracks in the superstructure of the wagons.

To create an accurate and reliable model for identifying cracks, the regions of interest in the images were annotated using a polygonal tool in the VGG Image Annotator. In the annotation process for the first database (Figure 5c), we outlined the regions containing cracks using the polygonal tool. This allowed the model to learn the characteristics and patterns associated with cracks in the superstructure.

For the annotation of the second database (Figure 5d), which includes areas with reported problems, two areas of interest were indicated using the polygonal tool. The first area of interest corresponded to the crack itself, while the second area of interest represented the wagon pillar. As shown in previous figures, a wagon contains multiple pillars where these cracks are commonly found. These areas experience significant variations in pressure and impact during the transportation and unloading of the ore.

By annotating the regions of interest accurately, the model could be trained to effectively detect and classify cracks in the superstructure of the wagons.

The mask R-CNN utilized in this study employed a network with pre-trained weights obtained from the MS COCO database [21]. This step was incorporated to enhance the training efficiency for the wagons present in the two databases, one with a single class and the other with two classes. In Figure 4, the hyperparameters of the tested network for creating the models are indicated.

To train the two databases, the dataset was divided into a 70% portion for training and a 30% portion for testing. This proportion ensured a suitable balance between training and evaluation. The training process consisted of 100 epochs, where each epoch represented a complete iteration through the training dataset.

The training was divided into 10 models. From these 10 models, the one exhibiting the best performance was selected to be evaluated using random images that were part of the 30% of images used in the testing stage. This evaluation step provided insights into the generalization capabilities of the selected model and its performance on unseen data.

In the first database, a total of 400 images were included. Among these, 140 images represented wagons with cracks, while 260 images depicted wagons without any cracking problems. Various parts of the wagons were considered in this database, including the initial and final sections, areas showing only the wagon’s plate, and regions displaying the supporting pillar.

The second database comprised 200 images that specifically focused on the most critical region, the area containing the supporting pillars. This database was balanced, consisting of 100 annotated images featuring wagons with cracks and pillars, along with 100 images containing only the supporting pillar class, as shown in the Figure 5c,d. Similarly to the first database, the division between training and testing followed a 70% training and 30% testing split.

For this work, the Mask R-CNN, a variant of the R-CNN architecture that incorporates instance segmentation was selected. The Mask R-CNN algorithm is capable of not only classifying objects in an image but also generating pixel-level masks to identify the precise regions associated with each object class [22].

In the context of this study, the Mask R-CNN algorithm was used to classify and color the regions of interest (such as cracks and supporting pillars) identified in the images. The algorithm assigns a certainty percentage to each class prediction, indicating the confidence level of the classification.

In the first stage, the model was trained on the images labeled with the cracked masks. This stage helped the model learn to generate accurate region proposals and refine them.

The second stage involved training the layers based on the ResNet network [23], which is a popular deep learning architecture known for its effectiveness in various computer vision tasks. The ResNet network used in this work had four stages, and the model learned to extract meaningful features from the input images at different levels of abstraction.

Finally, the third stage performed fine-tuning across all layers of the network. This stage helped in further refining the model’s ability to accurately detect and classify the regions of interest.

By leveraging the pre-trained weights and conducting multi-stage training, the model aimed to achieve better performance and accuracy in classifying and segmenting the regions of interest in the wagon images.

In the testing phase of the work, the remaining 30% of the dataset, which was not used for training, was used to evaluate the performance of the trained model. Each image from this test set was analyzed and classified individually, one at a time. The accuracy of the model in identifying the regions of interest (cracks or pillars) in the images was evaluated.

By applying this metric to the individual image classifications, this work could assess the overall effectiveness of the model in accurately identifying and classifying the cracks and pillars in the wagon images.

Figure 4 shows the information workflow, from collection to the final result of the Mask R-CNN. Data acquisition started with storing the video and pre-processing it into images. Within the preprocessing, the SSD detection algorithm was used, with the preparation and annotation of images into two different databases. For the execution of the Mask R-CNN, 10 different models were trained and 30 images were tested for each model.

#### 4.2.4. U-NET Methodology

U-NET is a neural network first designed to identify medical problems [24] in exam images, and its adaptation to other types of images was only a matter of time.

The U-NET used in this work used the same pre-processing performed for the creation of the two databases explained in the previous section. However, some particularities of the algorithm were implemented.

In addition to the structures of the training and testing images, the U-NET used in this work used another image called a mask; unlike Mask R-CNN, which has this annotation in a JSON file.

For the architecture of the U-NET network used in this work, two 3 × 3 convolutions were applied, each followed by a rectified linear unit (ReLU). This was followed by a 2 × 2 max pooling operation with two strides for downsampling.

With each downsampling step, the number of feature channels was doubled. Each step in the expanding path consisted of an upsampling of the feature map, followed by a 2 × 2 convolution that halved the number of feature channels, a concatenation with the correspondingly cropped feature map from the contracting path, and two 3 × 3 convolutions, each followed by a ReLU.

Cropping was necessary due to the loss of edge pixels in each convolution. In the final layer, a 1 × 1 convolution was used to map each 64-component feature vector onto the desired number of classes. In total, the network had 23 convolutional layers.

#### 4.2.5. Ensemble Methodology

Within the ensemble universe, the use of a parallel methodology was adopted for this work, so that a voting system was included in each of the classifiers. A voting classifier is a machine learning estimator that contains several training models or estimators to predict or classify based on grouping the results of each of the different models and using a certain metric, such as a simple average of the results.

Voting criteria can be in two forms: hard voting, in which the vote is calculated based only on the predicted class, or the second criterion soft voting, based on the probability of the result.

Figure 4 indicates four classifiers, each of which had a probability of being correct in two classes (1—if there are cracks and 2—without cracks). Then, an average was performed to identify the best results for the classifiers, with the average of class 1 being the best, so the ensemble voted for an image containing cracks.

In Figure 4, the same situation is indicated; however, only two classifiers were used for these tasks. Mask R-CNN and U-NET were the two classifiers in question.

## 5. Results

In this section, we will present the results found for the two types of databases. The first database contained 400 images with unbalanced classes. In this database, two classes were identified, and 35% of the images had problems. The second database consisted of only one class, but it contained 200 images, half of which had cracks. To present the accuracy results, we will use boxplot graphs for each test of the two databases.

### 5.1. Unbalanced Database Results for 2-Class Classification with Mask R-CNN Algorithm

Regarding the unbalanced database, an important observation about the results is the randomness of the images selected for testing. Out of the total 400 trained images, only approximately 35% of them exhibited irregularities or cracks. Based on this, 140 images were tested in 10 rounds, and in rounds 1, 2, and 9, none of the images captured by the algorithm yielded any results.

**Figure 7:**
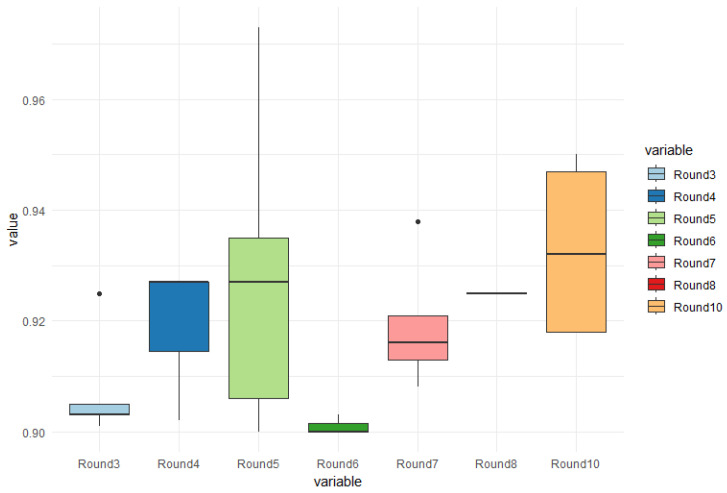
Boxplot graphs of the accuracy of cracking in train cars for unbalanced database using Mask R-CNN. Each round contained 30 random images, in the 10 rounds only 30% were composed of crack images. In round 5, 97.30% was the best result.

In this case, Figure 7 displays boxplot graphs depicting the accuracy of crack detection in the tested rounds. In all rounds, less than 50% of the images exhibited cracks. However, round 5 demonstrated the highest accuracy, achieving a hit rate of 97.30%, and for reasons illustrated in Table 1, it also displayed the best average accuracy among all rounds, in contrast to the number of test executions, with a value of 92.40%, indicating a greater variability in images in this round. Even though the highest result on average was achieved by Round 10 (93.33%).

With these results, we can verify that the imbalance of the database caused the problem of evasion of encounters with problems. In this case, a model with a balanced database is seen below, to identify if this problem continued.

To understand the results of the test round of the unbalanced database with two classes, with pillars and cracks being presented, Table 1 shows the number of images with cracks, the best result, and the average of their best hit rates, whether identifying a crack or not.

The use of two classes was an indication used to improve the identification of cracks, as these occur in the weld joints or folds of the wagon structure. In this case, the column sorts were disregarded in this round.

### 5.2. Balanced Database Results for 1-Class Classification with Mask R-CNN Algorithm

In the case of the balanced database, the accuracy was also used to evaluate the detection of cracks in freight trains. Similarly to the previous test, 10 rounds of tests were conducted, with each round consisting of 30 randomly chosen images from the total of 200 images used for training and testing. However, the specific results and accuracy metrics for the balanced database are not provided.

**Figure 8:**
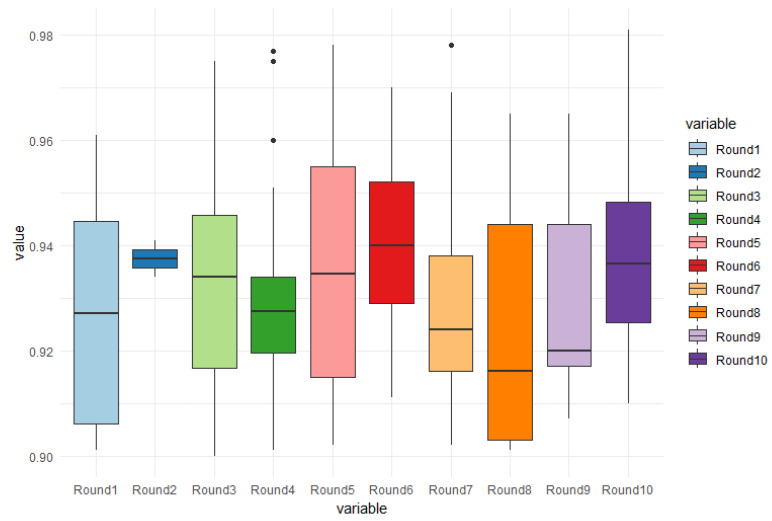
Boxplot graphs of accuracy of cracking in train cars using Mask R-CNN algorithm. Each round contained 30 random images; however, only in round 7 were problems found in all images tested, with an average of 92.29%.

Figure 8 displays the boxplot graphs depicting the accuracy results for the balanced database. It can be observed that in round 7, all thirty images tested showed cracking problems, resulting in the lowest accuracy value of 90.2%. The highest accuracy rate achieved was 97.80%, and the average accuracy across all rounds was 92.90%. In round 2, only two out of the thirty tested images showed results, which happened by chance and contributed to the highest accuracy rate of 94.41% for that particular round.

Among the 10 test rounds conducted, round 6 achieved the highest hit rate, with more than 70% of the images containing cracks. It identified 27 out of 30 images correctly, resulting in an average accuracy of 94.06%. The peak accuracy for round 6 reached 97%, as shown in the graph. However, it should be noted that both round 5 and round 7 achieved a higher hit rate of 97.80% for images with cracking problems, despite having a slightly lower overall average accuracy.

The test rounds that did not reach the threshold of 70% of images with cracking problems (rounds 1, 2, 9, and 10) are shown in Table 1, and it is worth noting that round 10 achieved the highest hit rate for an individual image, reaching 98.10% accuracy. Despite the lower overall number of images with problems, the average hit rate in round 10 was comparable to the rounds that had a higher proportion of images with cracks. This suggests that even in rounds with a lower prevalence of problems, the model was still able to accurately identify the presence of cracks in the tested images.

### 5.3. Applying U-Net and Ensemble on Unbalanced Databases for Crack Detection in Train Images

In this subsection, the results of the application of U-Net in its original architecture will be described. The model was tested on an unbalanced database consisting of 260 images without cracking problems and 140 images containing cracks.

For the first test presented in the next section, images with cracks were identified with U-NET. It is important to note that the U-Net model outperformed the Mask R-CNN model in this particular scenario (unbalanced database).

The ensemble methodology is a technique used to combine the predictions of multiple models in order to improve the overall performance and accuracy. In the context of this work, an ensemble approach was applied to the results obtained from the Mask R-CNN, U-Net, CNN, and multi-layer perceptron models, in order to enhance the success rates in identifying cracks in train car images.

By combining the predictions of multiple models, the ensemble approach aimed to leverage the strengths of each individual model and mitigate their weaknesses. This can lead to more robust and accurate results by reducing the impact of any individual model’s limitations or biases.

Common techniques for ensemble learning include majority voting, weighted voting, and stacking. These techniques could be applied to combine the predictions of the Mask R-CNN and U-Net models, potentially improving the overall accuracy and reliability of crack detection in train car images [25,26].

The results of an ensemble approach need to be evaluated and compared to the individual models’ performance to assess its effectiveness in improving the success rates. This can be done by measuring metrics such as accuracy [26].

Overall, the ensemble methodology applied in this work aimed to harness the collective power of multiple models to achieve better results in identifying cracks in train car images, ultimately enhancing the effectiveness of the crack-detection system.

#### 5.3.1. Unbalanced Database Results for 1 Class on U-NET

After dividing the database into training and test sets, with 260 images for training and 140 images for testing, the U-Net model was trained and evaluated. Several test runs were performed, and as a result, five models were generated for evaluation.

These five models were variations of the U-Net architecture trained on different meta parameter subsets for the same training data. By generating multiple models, the aim was to explore different representations and learn diverse patterns from the data, thereby increasing the chances of achieving better performance and accuracy.

**Figure 9:**
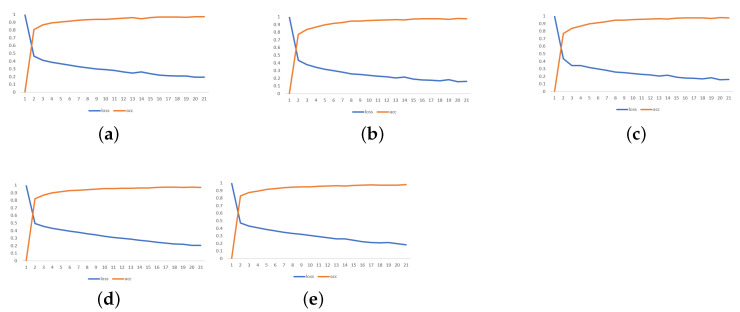
Training images of the five models for evaluation. (**a**) training 1. (**b**) training 2. (**c**) training 3. (**d**) training 4. (**e**) training 5 and best model.

Figure 9 illustrates the evaluation of loss and accuracy metrics for the five trained U-Net models over 20 epochs. The graph provides insights into the performance and learning progress of each model during the training process.

Based on the graph, it can be observed that all five models exhibited a similar trend in terms of loss and accuracy. As the number of training epochs increased, the loss decreased, indicating that the models were learning and improving their ability to minimize the difference between the predicted and actual values. At the same time, the accuracy increased, reflecting the models ability to correctly classify the input data.

Among the five models, model 5 (Figure 9e) stands out, as it achieved the highest accuracy and lowest loss during training. This suggests that this particular model learned to effectively capture and identify the patterns associated with cracks in the input images. Consequently, model 5 was selected for further testing and evaluation.

The decision to choose model 5 for testing was based on its superior performance and the fact that it had been trained on a larger number of images with cracking problems. This choice was expected to yield higher accuracy in detecting cracks in the test images, as indicated by its strong performance during training.

The evaluation of the selected U-Net model was carried out on a total of 60 randomly chosen images from the database. These images were divided into three different sets, each containing 20 images. The purpose of this evaluation was to assess the model’s performance in detecting cracks in various image samples.

**Figure 10:**
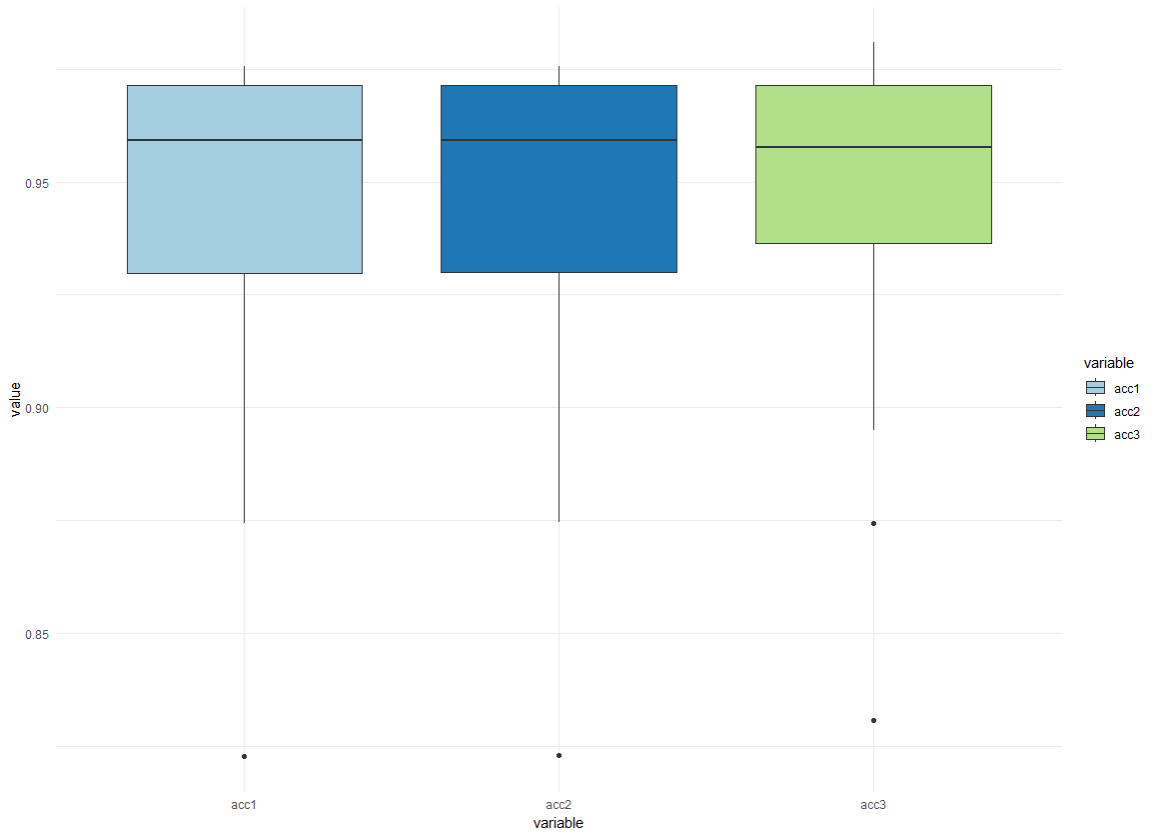
Precision *boxplot* of cracks in train cars. Each group contained 20 random images, in the best case an average of 94.44% was identified using U-NET.

In Figure 10, you can see the behavior of each of these groups of 20 images, divided into three different categories. The main observation from the figure is that the average hit rates for detecting cracks in the images were similar across the three groups. The first group had an average hit rate of 94.31%, the second group had an average hit rate of 94.32%, and the third group had an average hit rate of 94.44%.

In this context, as shown in Figure 10, it can be observed that this classification model did not require a large number of images for evaluation. Additionally, the success rate for the best model was around 97%, with the highest result being 98.08%.

#### 5.3.2. Ensemble with Unbalanced Database Results for 1-Class Classification

**Figure 11:**
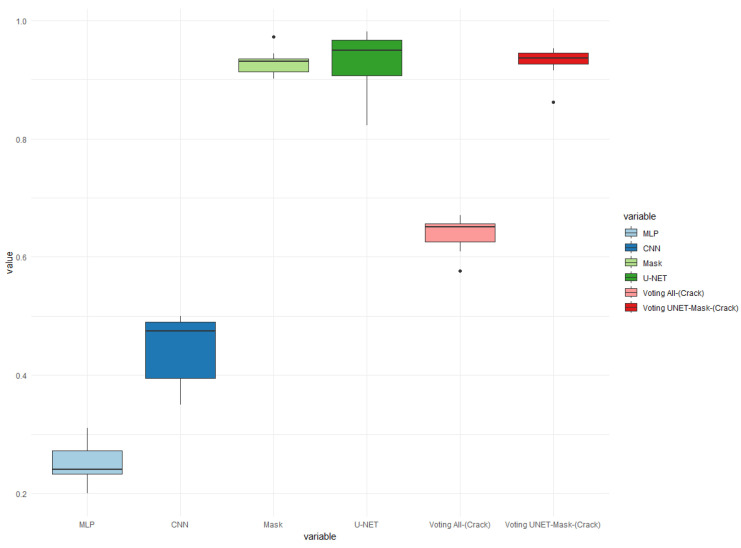
Precision boxplot charts of cracks in train cars for the best classifier models using an ensemble. Accuracy for 10 images in Test 1.

**Figure 12:**
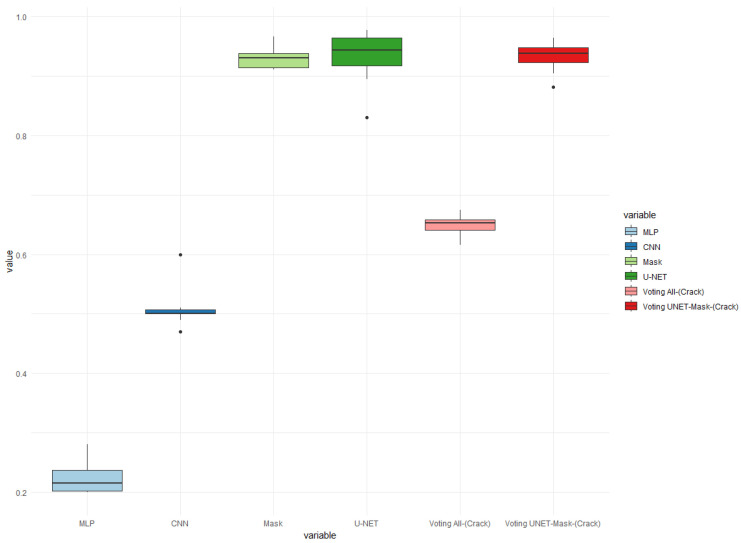
Precision boxplot charts of cracks in train cars for the best classifier models using an ensemble. Accuracy for 10 images in Test 2.

**Figure 13:**
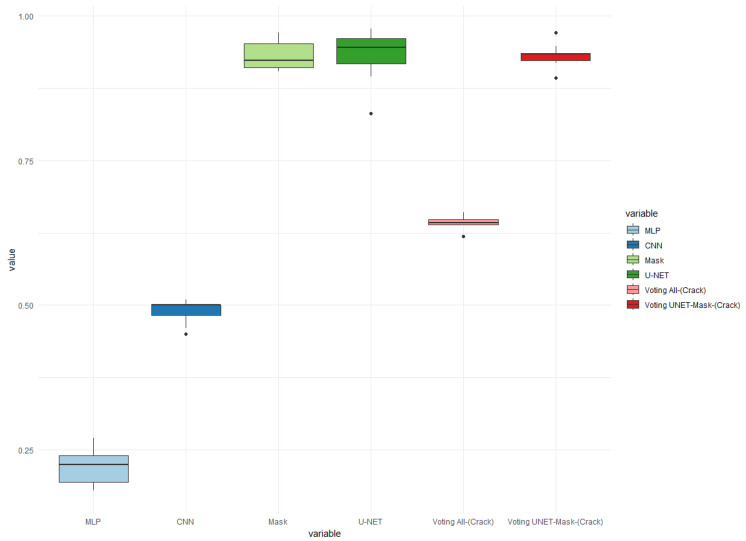
Precision boxplot charts of cracks in train cars for the best classifier models using an ensemble. Accuracy for 10 images in Test 3.

To further improve the results, the method of using an ensemble approach, based on a voting mechanism using accuracy, was adopted. In Figure 11, Figure 12 and Figure 13, the results for the three models of each of the four classifiers are presented. These models utilized a total of 400 images, with 260 images depicting train cars without any crack problems, and 140 images representing cars with cracks.

Furthermore, in Figure 11, it can be observed that 10 images per model were employed for testing. In each of these tests, apart from the four individual classifiers, an ensemble consisting of all four classifiers and a second ensemble comprising the Mask R-CNN and U-NET classifiers were also included.

The results depicted in Figure 11 indicate that the four classifiers achieved a maximum hit rate of 67% and an average of 63%. It can be observed that the use of an ensemble did not improve the performance of the individual classifiers. This can be attributed to the fact that the MLP classifier generated numerous errors when identifying cracked images.

When comparing the ensemble of four classifiers with the ensemble of two classifiers, a significant difference can be observed. This was mainly due to the Mask R-CNN and U-NET classifiers, which achieved remarkable results. The accuracy of the ensemble of four classifiers averaged around 92.97%, with the best case achieving a 95% accuracy rate on the images.

In Figure 12, a similar pattern is observed, where the ensemble of two classifiers performed better than the ensemble of four classifiers. The best hit rate in the two-class vote was 96%, which indicates an improvement compared to the results in test 1 of Figure 11. This improvement can be attributed to the two classifiers having a higher accuracy, with an average hit rate of 93.20%.

Similarly, Figure 13 confirms the previous observations, where the ensemble of two classifiers outperformed the ensemble of four classifiers. In the worst case scenario, the average hit rate was 64.37% and the best rate was 64.87%. However, in the best case scenario for the ensembles, the hit rate improved significantly, with a best rate of 97.03% and an average rate of 93.14%. This highlights the effectiveness of the ensemble approach in improving the overall accuracy of the classifiers.

The use of ensembles did not improve the results of the classifications performed by U-NET in checking cracks in train cars. However, the results did improve for the three tests compared to the average results of Mask R-CNN. The difference between their best results was less than 1%. This suggests that an ensemble approach may not be necessary for U-NET, as it already performs well on its own. However, for Mask R-CNN, the ensemble approach helped to improve its overall performance.

## 6. Previous Algorithm History and Discussion

To consider the Mask R-CNN method for crack classification activities in freight train cars, various techniques were explored. The approach involved experimenting with different types of neural networks, from simple ones to more complex convolutional neural networks, using various meta-parameters. The goal was to develop a solution that does not heavily rely on pre-processing and provides accurate results that are aligned with the expectations of the team responsible for verifying cracks in train wagons.

The decision not to rely on additional pre-processing techniques was primarily driven by the need for a fast and efficient system. The initial approach presented in this work, involving the use of complete 4K images, was deemed impractical, due to the computational burden and the inherent similarity between images. Since cracks in train cars are often small and localized, it was determined that dividing the images into smaller regions would be a more effective approach. This approach allowed for a more focused analysis of specific areas where cracks are likely to occur, resulting in improved classification accuracy.

The expert assessments revealed that other methods, such as using a common neural network with a softmax function, were not successful in achieving satisfactory results. One major challenge was the unrealistic classification time required by this approach. Additionally, the highly unbalanced nature of the database and the use of complete wagon images contributed to the low accuracy, with an average hit rate of only 25%. These limitations highlighted the need for a more efficient and focused approach, which led to the exploration of convolutional neural networks and the adoption of image division techniques, as discussed earlier.

The use of convolutional neural networks (CNNs) was explored extensively, considering various meta-parameters in an attempt to improve the results. However, the challenges posed by the high-resolution, unprocessed images and the similarity between the two classes limited the accuracy achieved. Table 2 provides an overview of the performance of the three different types of CNNs used in the search for an optimal methodology for the data and classification algorithm.

Unfortunately, the accuracy of these CNN models did not exceed 60%, indicating the complexity of the crack classification task and the need for alternative approaches. These unsuccessful attempts further motivated the development of the approach described in Section 4, which involved image division and the utilization of more specific algorithms like Mask R-CNN and U-NET.

**Figure 14:**
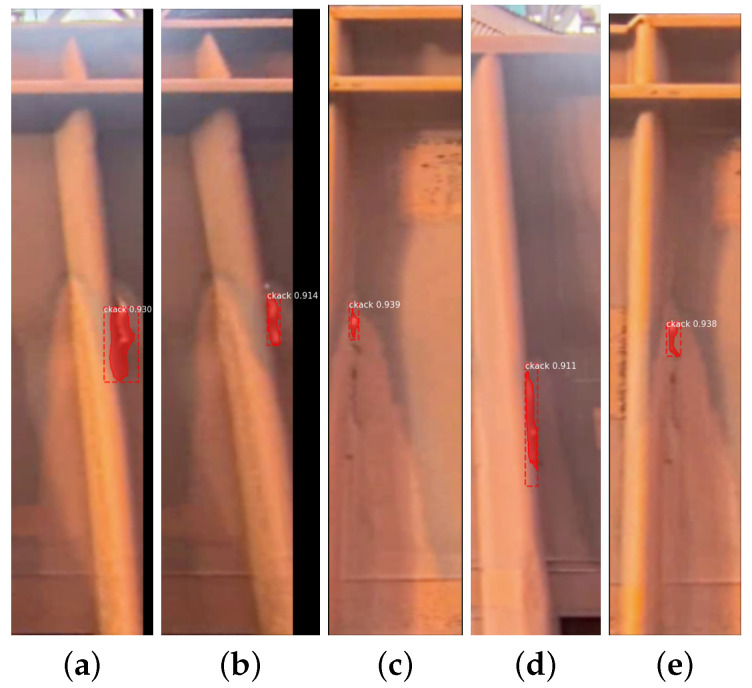
Result of the validation process on images for 5 different models. (**a**) Result 1. (**b**) Result 2. (**c**) Result 3. (**d**) Result 4. (**e**) Result 5.

The choice of using the Mask R-CNN algorithm for image preprocessing and tagging was based on its simplicity and effectiveness in handling image segmentation tasks. The algorithm’s ability to identify and mark specific regions (such as cracks) in images made it a suitable choice for the crack classification task at hand. In Figure 14, we can see examples of how the algorithm behaved at different times when evaluating the cracks in the trains, as well as its real confidence in the classification.

However, it is worth mentioning that there are other algorithms and approaches available for region-based image markup. Exploring alternative algorithms for region annotation and segmentation could be a potential avenue for future research and improvement in the crack classification process. Different algorithms may offer unique advantages or considerations based on the specific requirements and characteristics of the dataset and problem domain.

### Best Results Comparison

The best cases among the algorithms in the present study and the shortest testing times are presented in Table 3. It can be seen that training took the most time in the complete process; however, the testing time for each individual image was similar for all the selected algorithms.

The time values presented in Table 3 are a rough estimate, they show a round value, and this difference did not exceed 30 min. In an analysis in which we used time as the criterion for choosing the CNN implementation, Mask R-CNN and U-Net were more suitable for training. For the test response, the best case was the combination of ensemble quotation with Mask R-CNN and U-Net.

In neither case is the MLP algorithm recommended for deployment in production. In a crossover, the choice of the best result would be the Mask R-CNN algorithm, because when there is the need for retraining, it has the best results for time/accuracy. The issue of implementing such a system in a production environment to help employees identify defective parts would involve the company’s software approval process, as it is a computer system.

## 7. Conclusions and Future Work

The transportation of cargo plays a vital role in various industries, including mining. Trains are commonly used for transporting goods and raw materials from mining sites to processing facilities or distribution centers. The use of trains for this purpose offers several advantages, such as the ability to transport large quantities of cargo in a single trip and the ability to operate on established rail networks.

The use of computer vision and machine learning techniques for crack detection in freight trains is indeed a valuable contribution to the field. Traditional methods of crack detection, such as chemical tests and temperature tests, have their limitations and may not be suitable for continuous monitoring of train structures. The application of computer vision and machine learning algorithms can provide a non-destructive and automated approach to crack detection. By using cameras to capture images or videos of train structures, these algorithms can analyze visual data to identify and locate cracks or other anomalies.

Overall, the combination of computer vision and machine learning technologies holds great potential for improving the detection and monitoring of cracks in freight trains, contributing to the safety, maintenance, and overall performance of railway infrastructure.

Our paper differs from the works [6,12,16,17,18] cited in Section 2 of related works, firstly, because it deals with the identification of cracks in train car superstructures; some of those works reported the identification of defective parts, and others identified cracks, but not in train car superstructures and using real images. Next, no specific works of this nature were found, and our work differs from the other works. Certain studies have similarities, but not in the aspect of railway images. The work in [13,14] could be considered analogous in its data pre-processing for the identification of masks, but it used images in conventional format, not hyper-spectral and multi-spectral images.

The use of two databases with different characteristics provided a comprehensive approach to crack detection in freight trains. The first database consisted of 400 random images of pieces of wagons, containing two classes: images with cracks and images without cracks. However, this database was unbalanced, meaning that the number of images in each class was significantly different. This can pose challenges for classification models, as they may be biased towards the majority class. The second database contained 200 images with only one class, specifically images with annotated cracks. In this case, 50% of the images in the database contained annotated cracks. This database provided a focused and balanced set of images specifically designed to train and evaluate the classifier’s performance in crack detection.

The results obtained from the unbalanced database demonstrated the effectiveness of the classifier in detecting cracks in freight train images, even when the dataset was imbalanced. Despite only 30% of the images containing cracks being validated, the classifier achieved a high accuracy rate in its best result, reaching 98.08% with the U-NET algorithm. This indicates that the classifier was successful in identifying and classifying cracks in the tested images.

Moreover, the average accuracy of 92.40% across the testing rounds further confirmed the classifier’s ability to consistently perform well in detecting cracks. This average accuracy suggests that the classifier can reliably identify cracks in a majority of the tested images, contributing to the overall effectiveness of the crack-detection system.

It is worth noting that some models may not have found any images with cracks in their testing rounds. This could be attributed to the nature of the dataset and the specific characteristics of the images in those rounds. However, the high accuracy rates achieved in the best result and the average indicate that the classifier was capable of effectively detecting cracks in the majority of the tested images, showcasing its potential for crack detection for freight trains.

The results obtained from the balanced database further confirm the importance of a balanced dataset in achieving better classification performance for crack detection in freight trains. With a more balanced dataset, where approximately 65.66% of the tested images contained cracks (validation), the classifier demonstrated improved performance in identifying cracks across the 10 different models.

Furthermore, the use of a single objective class, focusing solely on identifying cracked wagons, proved to be effective for the Mask R-CNN classifier. By simplifying the classification task to a single class, the classifier was able to specifically focus on detecting and localizing cracks in the images, leading to improved accuracy in crack identification.

The best result achieved among the balanced models was an impressive 98.10% accuracy in one of the models. This indicates that the classifier was able to accurately identify and classify cracks in the majority of the tested images, showcasing its high precision in crack detection. Additionally, another model achieved the best average hit rate of 94.06%, demonstrating consistent performance across multiple testing rounds.

This work aimed to evaluate the effectiveness of the Mask R-CNN, U-NET, and ensemble classifier algorithms in identifying cracks in train cars. Two databases were utilized to conduct this evaluation, and the results demonstrated that both databases were successful in achieving the objective of crack detection.

However, it became evident that the use of a balanced database with a single class yielded better results in terms of the number of classified images and the learning capability of the algorithm. This balanced database consisted of images containing only the class “crack”, and it provided a more focused and targeted dataset for the classifier to learn from.

By utilizing a balanced database, where the distribution of images with cracks was more evenly represented, the classifier demonstrated improved performance in identifying and classifying cracks accurately. This dataset configuration allowed the algorithm to develop a better understanding of the features and characteristics associated with cracks, leading to more reliable and precise classification results.

The balanced database’s superiority can be attributed to several factors. Firstly, having a more balanced distribution of images ensured that the classifier had exposure to an adequate number of positive examples (cracked wagons), allowing it to learn the distinguishing features of cracks more effectively. Additionally, the absence of an overwhelming majority of negative examples (wagons without cracks) prevented the classifier from favoring the majority class, which can lead to biased and inaccurate results.

The better results achieved with the balanced database underscore the importance of dataset composition and balance in effectively training classifiers. It highlights the need to carefully consider dataset design and composition when developing machine learning models for crack detection in train cars or similar applications.

Overall, the use of a balanced database with a single class proved to be a more suitable approach for crack detection in train cars using the Mask R-CNN classifier algorithm. It resulted in improved performance, higher accuracy, and better learning capabilities, ultimately enhancing the effectiveness of the algorithm in identifying and classifying cracks accurately.

In summary, this work effectively answered the two questions posed in the introduction, proposing a new method for preparing image databases. It presents several approaches that establish a baseline for future research, as well as an investigation of various machine learning methods documented in the literature.

Future work in this area could explore the potential of ensemble methods combined with voting and pruning techniques, to further improve the classification of cracks in trains. Ensemble methods have shown promise in enhancing the performance of classifiers by combining the predictions of multiple models. By leveraging the diversity of different classifiers, an ensemble can often achieve better accuracy and robustness.

In the context of crack classification in trains, an ensemble approach could involve training multiple classifiers using different algorithms, architectures, or hyperparameter settings. These classifiers could then be combined through voting mechanisms, such as majority voting or weighted voting, to determine the final prediction. Additionally, pruning techniques could be employed to select a subset of the most informative and diverse classifiers for the ensemble, further improving the efficiency and performance.

## Figures and Tables

**Table 1:** Summary table of the number of images with problems found in each test round with the 10 different models created for the training. Results of the rounds of the tested algorithm for random selections in the databases.

Round	Quantity	Best Result	Average
1	0	X	X
2	0	X	X
3	5	0.925	0.9074
4	3	0.927	0.9183
5	13	**0.973**	0.924
6	3	0.903	0.9192
7	5	0.938	0.9192
8	1	0.925	0.925
9	0	X	X
10	4	0.95	0.933

**Table 2:** Results of runs with different models of CNNs in a database with 400 complete images of wagons divided into two classes, and with three different types of parameters in the CNN. CNN 1 passed one image at a time with a learning rate of 0.001; CNN 2 passed one image at a time with LR 0.0001; and CNN 3 ran with LR 0.001, but passing two images at a time in the batch. Values for the initial rounds that served to evolve the algorithms used.

Round	CNN 1	CNN 2	CNN 3
1	0.38	0.5	0.5
2	0.35	0.5	0.5
3	0.41	0.5	0.5
4	0.49	0.49	0.5
5	0.5	0.51	0.46
6	0.5	0.5	0.45
7	0.47	0.5	0.49
8	0.49	**0.6**	0.5
9	0.48	0.47	0.51
10	0.39	0.51	0.48

**Table 3:** Comparison of all algorithms containing the best results, regardless of the database used and the best times for these models.

Algorithm	Best Result	Training Time	Testing Time
MLP	25%	5 h	360 s
CNN	60%	2 h	60 s
**Mask R-CNN**	**98.10%**	**2 h**	60 s
U-NET	98.08%	2 h	60 s
Ensemble—4	67%	7 h	40 s
Ensemble—2	97.07%	7 h	**30 s**

## Data Availability

Embargo on data due to commercial restrictions.
